# Early identification of NORSE and transfer to care setting with appropriate supports: A proposed algorithm

**DOI:** 10.3389/fneur.2023.1072020

**Published:** 2023-04-11

**Authors:** Sarah A. Vinette, Gordon Bryan Young, Houman Khosravani

**Affiliations:** ^1^Division of Neurology, Department of Medicine, University of Toronto, Toronto, ON, Canada; ^2^Grey Bruce Health Services, Owen Sound, ON, Canada; ^3^Hurvitz Brain Sciences Program, Neurology Quality and Innovation Lab, Division of Neurology, Department of Medicine, Sunnybrook Health Sciences Center, University of Toronto, Toronto, ON, Canada

**Keywords:** NORSE, FIRES, status epilepticus, epilepsy, neurocritical care

## Abstract

New-onset refractory status epilepticus (NORSE) is a clinical presentation where an individual develops refractory status epilepticus without active epilepsy, or related neurological conditions. A subset of these individuals has a preceding fever and would be diagnosed with febrile infection-related epilepsy syndrome (FIRES). The underlying etiology of this condition varies and includes autoimmune and viral encephalitides. These conditions require multiple specialized health care teams working collaboratively and specific resources for investigation of the underlying etiology and management to provide optimal patient care. In this paper, we provide: (1) recommendations upon early recognition of NORSE and FIRES, (2) guidance on the resources needed to optimally provide care, and (3) guidance on considerations to initiate transfer of patients to a more specialized medical center. Additional recommendations for resource-austere centers without the ability to transfer such patients are also discussed. These recommendations are only for adult patients with NORSE as pediatric patients may require additional special considerations.

## 1. Introduction

New-onset refractory status epilepticus (NORSE) can be defined as a clinical presentation in a patient without active epilepsy or other existing relevant neurological disorder, with new onset of refractory status epilepticus in the absence of a clear acute or active structural, metabolic, or toxic cause. Refractory status epilepticus (RSE) is a condition where continuous or recurrent seizures do not stop with standard anti-epileptic medications (1). The duration of seizure activity required to diagnose status epilepticus varies by type of seizure. Generalized convulsive status epilepticus involves at least 5 min of continuous seizure activity or repeated seizures without return to baseline in-between lasting at least 5 min. The timepoint at which this prolonged seizure activity may result in long term consequences is believed to be at 30 min. Focal status epilepticus is defined by 10 min or more of focal seizure activity with impaired awareness, with the possibility for long term consequences to arise after 60 min. Nonconvulsive status epilepticus occurs when seizure activity is present for 10 or more minutes lacking prominent motor symptoms (2). Status epilepticus is considered refractory if “persisting despite administration of at least 2 appropriately selected and dosed parenteral medications including a benzodiazepine. There is no specific seizure duration required” (1). NORSE has a subset of cases meeting the definition for febrile infection-related epilepsy syndrome (FIRES), where a febrile illness precedes the onset of refractory status epilepticus by 24 h to 2 weeks (1).

Although the prevalence of NORSE has not been studied and identified, the annual incidence of refractory status epilepticus is estimated to be 3.0–7.2 per 100,000 adults per year (3, 4). One study showed 20% of cases of refractory status epilepticus did not have a clear etiology after initial investigations (5), thereby making NORSE a rare condition. Despite its incidence, there is significant associated morbidity and mortality (5), and thus early recognition, identification, and transfer to an appropriate care-setting are paramount.

## 2. Early recognition

A prodromal period can precede the onset NORSE by a couple of weeks and often includes non-specific symptoms such as confusion, fever, fatigue, headache, gastrointestinal, or respiratory symptoms. It is estimated that this prodromal period is present in ~60% of cases (5). Individuals may then develop infrequent seizures which evolve into status epilepticus (6).

NORSE should be a diagnostic consideration in individuals presenting with new onset recurrent seizures evolving into status epilepticus with no known history of epilepsy and no clear identifiable etiology after initial blood work, CSF studies, and brain imaging has been completed.

There are several identified predictors of prolonged refractory status epilepticus. These include the presence of acute brain lesions, increased severity of status epilepticus (measured using the status epilepticus severity score [STESS] (7)), non-convulsive status epilepticus with coma, and increased serum albumin levels at onset of status epilepticus (8). STESS alone was found to be predictive of outcome and includes age, history of seizures, seizure type, and degree of impaired consciousness (7). The presence of these predictors may serve as a flag for clinicians, as each day of status epilepticus is associated with increased risk of mortality (8). As these predictive markers were identified in a more general population of individuals with status epilepticus, is unclear if they hold similar predictive value in the subset of those with NORSE and FIRES. It is plausible that considering NORSE top-of-mind in the differential diagnosis of new status epilepticus may aid in early recognition and clinical considerations for transfer, especially in resource-austere settings.

## 3. Investigations and treatment requirements

While it is of prime importance to provide airway and cardiovascular/blood pressure support and to halt seizures as soon as possible to prevent further neurologic injury, further consideration must be given to other aspects, including monitoring for breakthrough or nonconvulsive seizures and searching for an underlying etiology; especially one that can be treated. Once the patient is reasonably stabilized, consideration should be given to transferring the patient to a tertiary care center where more specialized investigations and care can be implemented.

An important aspect of a higher-level of care includes the multidisciplinary team, and ability to case conference about such patients with actionable diagnostic and treatment strategies. Centers with neurocritical care expertise are also uniquely qualified to monitor and treat these complex patients. Consultant teams are essential for the co-management of these cases. These services include Neurology with expertise in epilepsy and Internal Medicine and its subspecialties, who may be needed to help manage the multi-system effects of prolonged seizure activity and complications of antiseizure treatments including anesthetics (9). Individuals who continue to have seizures refractory to available therapies or sustain significant complications benefit from early involvement of palliative care for symptom management, bereavement support for the patients’ family, and possibly end of life care (10–12).

Furthermore, specialized neuro-focused centers play an important role in the recovery phase where multidisciplinary team members such as physiotherapy, occupational therapy, speech language pathology, and rehabilitation medicine can collaboratively address the ongoing needs of patients who survive the acute phase of this illness. The burden of critical illness on NORSE patients is an important consideration with individuals exhibiting prolonged hospital stays, with a median ICU stay of 26 days (13, 14), often with neurological sequalae including altered cognition and development of epilepsy (12, 15, 16).

From an investigation standpoint, readily available access to continuous scalp EEG monitoring with video and MR imaging are important aspects of care. Having access to specialized diagnostic tests including autoimmune, paraneoplastic antibody testing, viral PCR testing may aid in early diagnosis (or lack thereof) of underlying etiology. In a significant proportion of cases, no etiology is identified, rendering their classification as cryptogenic (5), although with increasing recognition of various autoimmune etiologies the cryptogenic category is diminishing. In addition to standard treatment, some patients with NORSE may benefit from immunomodulatory therapies, such as corticosteroids, intravenous immunoglobulins, or plasma exchange, particularly if the underlying cause is thought to be autoimmune or inflammatory. The provision of some of these services (e.g., plasma exchange) is a challenge in austere settings.

In resource-austere settings where transfer to a comprehensive center is not possible, substitution of modalities of investigation and/or virtual care may be considered. Critical care settings without subspecialized expertise, CT imaging instead of MRI to rule out gross structural lesions, and serial routine EEGs instead of continuous monitoring can be considered (Table 1).

**Table 1:** Resources needed to provide optimal care for NORSE patients in the acute phase of their illness.

Well-resourced settings	Recommended timing after seizure onset	Substitutions for consideration in resource-austere settings
Medical services and healthcare professionals		
Critical care specialists with neurocritical care expertise	Immediate	Critical care specialists
Neurologically-trained nurses and house staff	Immediate	
Neurologists with expertise in epilepsy	Within 24 h	Experienced Neurologists
Internal medicine subspecialty services (Rheumatology, Immunology, Gastro-enterology, Nephrology, Cardiology)	As needed. Variable timing of initiation guided by any relevant rheumatologic/immunologic findings on investigations, complications that may arise during hospital course, and immunologic therapies considered	Dedicated anesthetists
Palliative care –neurology specific	As needed. Variable timing of initiation guided by the need for reevaluating goals of care, enhancing focus on comfort care, and/or supporting patient’s family and healthcare team	Palliative care. Recommend expert consultation, virtual or with transfer of care
Management		
Securing airway and hemodynamic stabilization	Immediate (0–5 min)	
Parenteral anti-seizure and anesthetic medications, inhalational anesthesia, hypothermia	First line (benzodiazepine): 5–15 min Second line: 20–40 min Third line: 40–60 min	Anti-seizure medications administered *via* alternative routes (PR, IM, NG tube) or intravenously
Consider empiric antibiotic and antiviral coverage (e.g., for HSV)	Within 24 h	
Immune-modulating/suppressive therapies	Consider within 72 h, with expert consultation, with first-line treatment of IV methylprednisolone or IVIG	Recommend expert consultation, virtual or with transfer of care
Ketogenic diet	Consider within the first week. The earliest the ketogenic diet can be considered in (S)RSE is after failure of first-and second-line antiepileptic drugs	Recommend expert consultation, virtual or with transfer of care
Monitoring for complications, (e.g., hypotension, ileus, pneumonia)	Continuous throughout ICU admission	
Investigations		
Initial blood work, including: -CBC, electrolytes (with extended electrolytes), creatinine, liver enzymes. -Point of care glucose -Toxicology screen -Blood cultures	Immediate (0–5 min)	
Additional blood work, including: -Viral and bacterial serologies -Autoimmune panel: ANA, ANCA, Anti-thyroid, anti-neuronal surface antigens -Paraneoplastic antibody panel	Within 24 h.	
MRI brain with contrast	Within 48 h.	CT head
Continuous EEG monitoring, automated EEG, preferably with video	Within 24 h.	Repeated routine EEGs
Lumbar puncture with CSF testing for: -Cell count, protein, glucose, lactate, viral PCR, bacterial and fungal culture, cytology, autoimmune and paraneoplastic panels	Within 48 h	
If ongoing seizure activity, recommended transfer timepoints:		
Transfer within:	If needed to obtain:	
24 h	MRI brain, EEG	
48–72 h	Continuous EEG monitoring, neurocritical care expertise	
72 h and beyond	Immunosuppressive treatments (e.g., IVIG, plasmapheresis, anakinra), ketogenic diet	

The final column outlines possible substitutions in resource-limited settings.

HSV, herpes simplex virus; IV, intravenous; IVIG, intravenous immune globulin; CBC, complete blood count; ANA, antinuclear antibody; ANCA, antineutrophil cytoplasmic antibodies; MRI, magnetic resonance imaging; EEG, electroencephalogram; CSF, cerebrospinal fluid; PCR, polymerase chain reaction; RSE, refractory status epilepticus; SRSE, super refractory status epilepticus. References: (13, 17–20).

## 4. Proposed algorithm for recognition and transfer

When a patient is identified as having ongoing seizure activity, beyond the timepoints required for meeting criteria of status epilepticus (2), and after administration of an appropriately dosed parenteral benzodiazepine and another appropriate medication for treating status epilepticus (1) a diagnosis of refractory epilepticus is made. Specialized critical care services are crucial for providing optimal care to patients with NORSE, as this condition requires prompt and aggressive treatment in an ICU setting. Early transfer is also important because the critical care management of NORSE patients has potentially life-threatening complications that can arise such as respiratory failure (severe acute respiratory distress syndrome [ARDS]), cardiac complications (from seizure or anesthetics), gastro-intestinal complications, and metabolic disturbances.

When transferring a patient with NORSE to a specialized critical care facility, special considerations with attention to airway patency, adequate circulatory support, and provision of seizure-stabilizing medications and equipment for transport are necessary. A trained medical team with expertise in critical care and neurology should oversee the transfer, with appropriate monitoring and interventions in place to manage any potential complications that may arise during transport.

**Figure 1:**
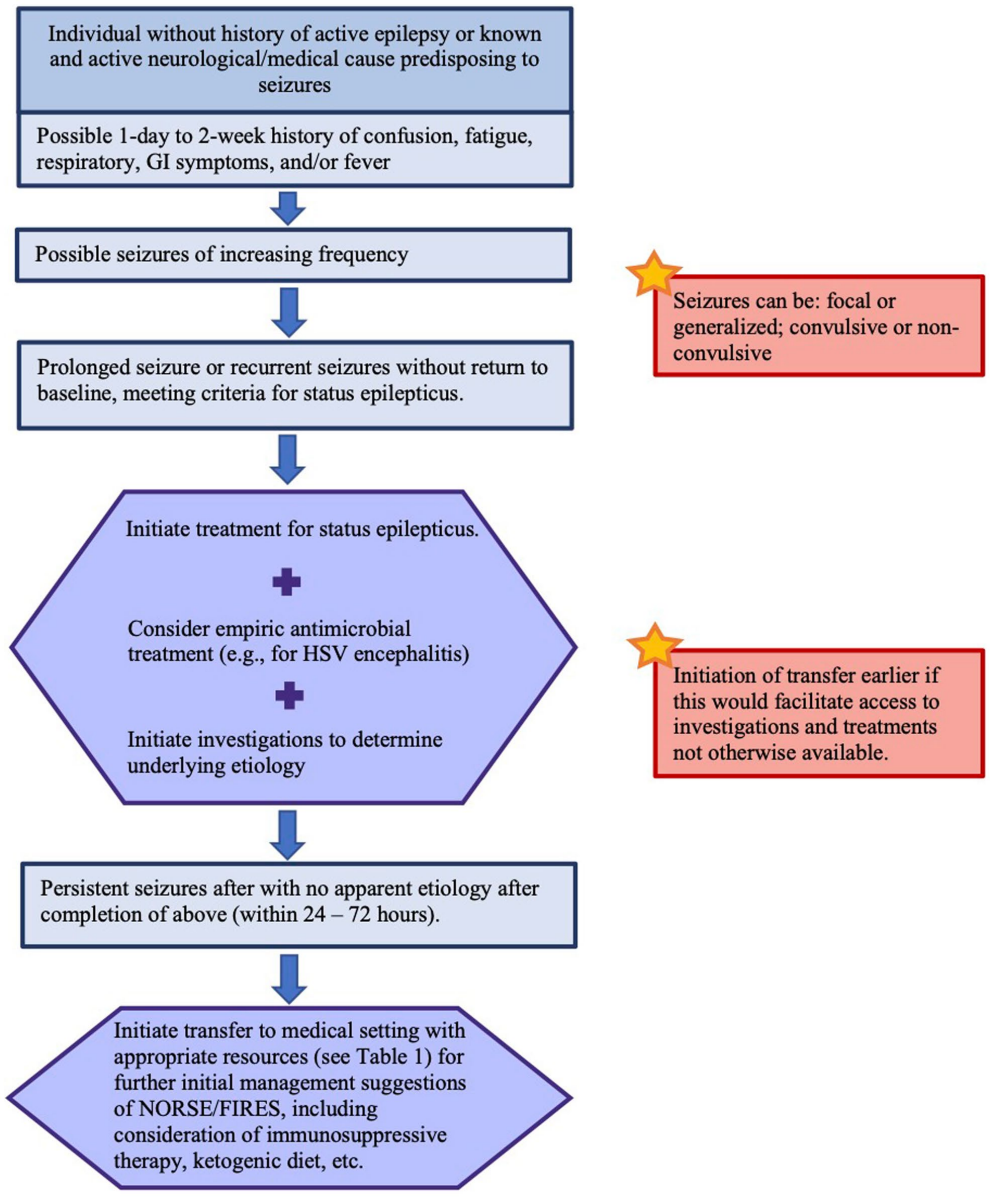
Proposed algorithm for recognition of NORSE/FIRES and transfer to a medical setting with appropriate resources. Please refer to reference (13) for details on recommended management of status epilepticus. Please refer to references (17, 20) for detailed recommendations on investigations and management of NORSE/FIRES.

Early transfer should be facilitated, when possible, especially in cases where a transfer would enable access to essential tests (e.g., MRI, cEEG, neurocritical care, use of volatile anesthetics in the ICU for sedation or seizure suppression), and neuromodulatory treatment options (IVIG, plasma exchange, biologic treatments). Early transfer is especially important when nonconvulsive status epilepticus is suspected or there is lack of access to routine and serial EEG given the morbidity and mortality associated with prolonged status epilepticus (8). See Figure 1 for a proposed algorithm for identification and early transfer (13, 17).

## 5. Conclusion

Overall, early identification of NORSE and associated conditions such as FIRES is essential to initiate appropriate investigations and management. Optimal patient care involves a multidisciplinary approach and numerous investigations and treatments. We advocate for early transfer to a specialized center with these resources with the aim of mitigating the known risks and downstream complications of NORSE. These recommendations pertain to adult patients as pediatric patients may require additional special considerations.

## Data availability statement

The original contributions presented in the study are included in the article/Supplementary material, further inquiries can be directed to the corresponding author.

## Author contributions

HK conceived this manuscript and oversaw direction and planning. SV wrote the first draft of this manuscript in consultation with HK and GY. All authors contributed to the content of this manuscript and were involved in editing this work.

## Funding

This work was supported by the University of Toronto.

## Conflict of interest

The authors declare that this work was conducted in the absence of any commercial or financial relationships that could be construed as a potential conflict of interest.

## Publisher’s note

All claims expressed in this article are solely those of the authors and do not necessarily represent those of their affiliated organizations, or those of the publisher, the editors and the reviewers. Any product that may be evaluated in this article, or claim that may be made by its manufacturer, is not guaranteed or endorsed by the publisher.
